# Cardiovascular Risk Factors and the Severity of COVID-19 Disease

**DOI:** 10.7759/cureus.15486

**Published:** 2021-06-07

**Authors:** Imane Motaib, Saad Zbiri, Saloua Elamari, Amal Haoudar, Asma Chadli, Chafik El Kettani

**Affiliations:** 1 Endocrinology, Diabetes, and Metabolism, Mohammed VI University of Health Sciences (UM6SS), Casablanca, MAR; 2 Epidemiology and Public Health, Laboratory of Public Health, Health Economics, and Health Management, International School of Public Health, Mohammed VI University of Health Sciences (UM6SS), Casablanca, MAR; 3 Endocrinology, Diabetology, Metabolic Disease, and Nutrition, Mohammed VI University of Health Sciences (UM6SS), Casablanca, MAR; 4 Anesthesia and Critical Care, Cheikh Khalifa International University Hospital, Mohammed VI University of Health Sciences (UM6SS), Casablanca, MAR

**Keywords:** traditional cardiovascular risk factors, covid 19, severity markers, sar-cov 2 infection

## Abstract

Background and objective

Several cardiovascular risk factors have emerged as important determinants of severe illness and death among coronavirus disease 2019 (COVID-19) patients. However, the full impact of these cardiovascular risk factors is still under investigation. This study aimed to investigate the association between patients' level of cardiovascular risk and the severity of COVID-19.

Materials and methods

This observational study included all adult patients with COVID-19 hospitalized at Sheikh Khalifa Ibn Zaid International University Hospital from March 20 to May 10, 2020. The cardiovascular risk level was assessed by the doctor responsible for each patient based on the 2019 European Society of Cardiology (ESC), the European Atherosclerosis Society (EAS), and the European Association for the Study of Diabetes (EASD) guidelines. We examined the association between the patients' level of cardiovascular risk and their severity of COVID-19 disease by using a logistic regression model.

Results

Among 133 patients with confirmed COVID-19, 46.6% had a low cardiovascular risk level, 19.5% had a moderate risk level, 15.8% had a high risk level, and 18.1% was found to have a very high risk level. Patients with different cardiovascular risk levels had significantly different rates of complications including secondary infection (p-value: <0.001), acute respiratory distress syndrome (ARDS) (p-value = 0.017), intensive care unit (ICU) admission (p-value: <0.001), and death (p-value: <0.001). A patient's very high cardiovascular risk level versus low, moderate, or high cardiovascular risk level was independently associated with ICU admission [OR = 6.42, 95% CI: (1.45-28.30)].

Conclusion

Based on our findings, an increased level of cardiovascular risk among patients was strongly associated with the severity of COVID-19. This study also highlights the need for assessing cardiovascular risk factors in all patients with COVID-19.

## Introduction

The novel coronavirus disease 2019 (COVID-19) was first identified in Wuhan, China in December 2019, and since then it has spread rapidly around the world. The World Health Organization (WHO) subsequently declared COVID-19 as a global pandemic on March 11, 2020 [1]. As of April 14, 2021, there have been 136,996,364 confirmed cases of COVID-19 globally, which have resulted in 2,951,832 deaths [2].

Previous studies on the condition have observed that elderly patients and those with comorbidities such as diabetes, hypertension, and cardiovascular diseases are more likely to develop severe forms of COVID-19, with more complications and/or more susceptibility to death from the disease [3,4]. However, the impact of these cardiovascular risk factors such as age, hypertension, diabetes, and cardiovascular diseases on the clinical outcomes and the prognosis of COVID-19 disease is still poorly understood.

This study investigated the association between the level of the patients' cardiovascular risk and the severity of COVID-19. We hypothesized that patients with increased cardiovascular risk levels, compared to those with a lower level of cardiovascular risk, are more likely to develop severe forms of COVID-19.

## Materials and methods

Study design

This single-center observational study included all adult patients (aged 18 years and above) admitted with confirmed COVID-19 disease to the Sheikh Khalifa Ibn Zaid International University Hospital in Casablanca, Morocco, between March 20 and May 10, 2020. This hospital has been mandated by the Moroccan Ministry of Health to care for patients with COVID-19. Depending on the severity of COVID-19, patients were admitted to the intensive care unit (ICU) (severe patients) or provided non-ICU care (non-severe patients). Criteria of ICU admission were defined based on the WHO interim guidelines: respiratory rate of >30 breaths per minute, severe respiratory distress or oxygen saturation level of SpO_2_ below 92% with 4 l of oxygen ventilation, or neurological or hemodynamic disorders [5]. Pregnant women and patients under the age of 18 were excluded from the study.

Data collection

Data were collected from the electronic medical records of patients by a trained team of physicians. These medical investigators gathered and reviewed the data of all patients.

For each patient, we collected data related to her/his cardiovascular risk level as well as demographic characteristics including age and gender; comorbidities including hypertension, diabetes, cardiovascular diseases (atherosclerotic cardiovascular disease, heart failure, arrhythmia, etc.), respiratory diseases (chronic obstructive pulmonary disease, asthma, etc.), dyslipidemia, and other diseases and risk factors (hyperuricemia, neoplasia, smoking, obesity, etc.); clinical symptoms including fever, general symptoms (dizziness, myalgias, asthenia, etc.), respiratory symptoms (dyspnea, cough, etc.), as well as ear, nose, and throat (ENT) and digestive symptoms; and clinical outcomes including intensive care unit (ICU) admission, invasive mechanical ventilation use, the onset of complications [acute respiratory distress syndrome (ARDS), secondary infection, multiple organ failure, thromboembolic disease], and deaths.

Outcomes definition

The diagnosis of COVID-19 was based on the WHO interim guidelines [6] and confirmed as per reverse transcription-polymerase chain reaction (RT-PCR) assays. Diabetes was defined as a self-reported medical history of diabetes. Newly diagnosed diabetes was defined based on the American Diabetes Association (ADA) criteria [7], including fasting plasma glucose of over 126 mg/dl and/or random glycemia higher than 200 mg/dl and classic signs of hyperglycemia and/or HbA1c of ≥6.5% during the hospital stay. Hypertension was defined as blood pressure above 140/90 mmHg, and ARDS was defined according to the Berlin definition [8].

Outcomes assessment

A cardiovascular risk assessment was performed for each patient by the doctor who had recommended her/his hospitalization. As shown in Table 1, doctors at the study hospital performed the cardiovascular risk stratification based on the 2019 European Society of Cardiology (ESC) and European Atherosclerosis Society (EAS) guidelines, and for patients with diabetes, as per the 2019 ESC and the European Association for the Study of Diabetes (EASD) guidelines [9,10]. This cardiovascular risk stratification is commonly used in many countries including Morocco [11]. Based on this, patients were classified into four cardiovascular risk level categories: very high risk level, high risk level, moderate risk level, and low risk level.

**Table 1:** Cardiovascular risk level categories* *[9,10] ^a^Target organ damage is defined as microalbuminuria, retinopathy, or neuropathy ASCVD: atherosclerotic cardiovascular disease; BP: blood pressure; CKD: chronic kidney disease; CVD: cardiovascular disease; DM: diabetes mellitus; eGFR: estimated glomerular filtration rate; FH: familial hypercholesterolemia; LDL-C: low-density lipoprotein cholesterol; SCORE: Systematic Coronary Risk Estimation; T1DM: type 1 DM; T2DM: type 2 DM; TC: total cholesterol

Risk level	Description
Very high risk level	Documented ASCVD DM with target organ damage^a^, or at least three major risk factors, or early onset of T1DM of long duration (>20 years). Severe CKD (eGFR of <30 mL/min/1.73 m^2^). A calculated SCORE of >10% for 10-year risk of fatal CVD. FH with ASCVD or another major risk factor
High risk level	Markedly elevated single risk factors, in particular TC of >8 mmol/L (>310 mg/dL), LDL-C of >4.9 mmol/L (>190 mg/dL), or BP of >180/110 mmHg. Patients with FH without other major risk factors. Patients with DM without target organ damage^a^, with DM duration of >10 years, or another additional risk factor. Moderate CKD (eGFR of 30-59 mL/min/1.73 m^2^). A calculated SCORE of >5% and <10% for 10-year risk of fatal CVD
Moderate risk level	Young patients (T1DM: <35 years; T2DM: <50 years) with DM duration of <10 years, without other risk factors. Calculated SCORE of >1% and <5% for 10-year risk of fatal CVD
Low risk level	Calculated SCORE of <1% for 10-year risk of fatal CVD

Ethical considerations

The study was approved by the Institutional Ethics Board of the Sheikh Khalifa Ibn Zaid International University Hospital (approval number: CE_UM6SS/1/06/2020 - April 3, 2020). Patient consent was waived as the study included only unidentifiable data, in accordance with the national law.

Statistical analysis

For descriptive analysis, we presented continuous variables as medians with interquartile ranges (IQRs) (small sample) and categorical variables as percentages. Patients with different cardiovascular risk level categories were compared in terms of demographics characteristics, comorbidities, clinical symptoms, and clinical outcomes, using the nonparametric k-sample test on the equality of medians for continuous variables and using the Fisher's exact test for categorical variables.

Secondly, univariate analysis was performed to study the association between all available variables, as described above, and ICU admissions. Finally, multivariate logistic regression was performed to examine the association between cardiovascular risk level and ICU admissions. Since cardiovascular risk might be highly correlated with many variables, particularly those related to comorbidities, we used a stepwise multivariate model based on a bidirectional elimination, with a p-value of <0.05 and including all significant control variables as found in the univariate analysis. Results were reported as odds ratios (ORs) with their 95% confidence intervals (CIs).

All statistical analyses were performed using the STATA software (StataCorp LLC, College Station, TX). P-values were two-sided, and those <0.05 were considered statistically significant.

## Results

A total of 133 adult patients who were hospitalized at Sheikh Khalifa Ibn Zaid International University Hospital with confirmed COVID-19 infection from March 20 to May 10, 2020, were included in the study. Table 2 presents the characteristics of the study population. The median age of included patients was 53 years (IQR: 36-64 years); 55.6% of the included patients were male. The most common cardiovascular risk factors were hypertension (28.6%), diabetes (14.3%), and preexisting cardiovascular disease (13.5%). Among the study population, 46.6% had a low cardiovascular risk level, 19.5% had a moderate cardiovascular risk level, 15.8% had a high cardiovascular risk level, and 18.1% had a very high cardiovascular risk level.

**Table 2:** Characteristics of the study population ^a^Dizziness, myalgias, asthenia, etc. ^b^Hyperuricemia, neoplasia, smoking, obesity, etc. ENT: ear, nose, and throat; ARDS: acute respiratory distress syndrome; ICU: intensive care unit; IQR: interquartile range

Variables	Values
Demographics	
Age, years, median (IQR)	53 (36-64)
Male gender, n (%)	74 (55.6)
Cardiovascular risk, n (%)	
Low level	62 (46.6)
Moderate level	26 (19.5)
High level	21 (15.8)
Very high level	24 (18.1)
Comorbidities, n (%)	
Hypertension	38 (28.6)
Diabetes	19 (14.3)
Cardiovascular disease	18 (13.5)
Respiratory disease	11 (8.3)
Dyslipidemia	11 (8.3)
Other diseases^a^	16 (12)
Clinical symptoms, n (%)	
Fever	60 (45.1)
General symptoms^b^	55 (41.4)
Respiratory symptoms	77 (57.9)
ENT symptoms	40 (30.1)
Digestive symptoms	32 (24.1)
Outcomes, n (%)	
Secondary infection	27 (20.3)
Invasive mechanical ventilation	14 (10.5)
ARDS	13 (9.8)
Thromboembolic complication	4 (3)
Multi-organ failure	9 (6.8)
ICU admission	46 (34.6)
Death	14 (10.5)

The characteristics of the study population as per their cardiovascular risk levels are presented in Table 3. Patients with different cardiovascular risk levels were comparable in terms of clinical symptoms (p-value: >0.05), but significantly differed in terms of demographics including age (p-value: <0.001) and gender (p-value = 0.028), in terms of comorbidities including hypertension (p-value: <0.001), diabetes (p-value: <0.001), cardiovascular disease (p-value: <0.001), dyslipidemia (p-value: = 0.008), and other diseases (p-value = 0.041), and in terms of clinical outcomes including secondary infection (p-value: <0.001), requirement for invasive mechanical ventilation (p-value = 0.001), ARDS (p-value = 0.017), ICU admission (p-value: <0.001), and death (p-value: <0.001). However, there were no significant differences regarding thromboembolic complications and multi-organ failure (p-values of 0.743 and 0.148, respectively). Notably, patients with a very high cardiovascular risk level also had higher rates of secondary infections, invasive mechanical ventilations, ARDS, ICU admissions, and deaths, when compared to patients with lower levels of cardiovascular risk (low, moderate, or high level of cardiovascular risk).

**Table 3:** Characteristics of the study population according to their cardiovascular risk level ^a^Dizziness, myalgias, asthenia, etc ^b^Hyperuricemia, neoplasia, smoking, obesity, etc. ENT: ear, nose, and throat; ARDS: acute respiratory distress syndrome; ICU: intensive care unit; IQR: interquartile range

Variables	Low risk level (n = 62)	Moderate risk level (n = 26)	High risk level (n = 21)	Very high risk level (n = 24)	P-value
Demographics					
Age, years, median (IQR)	36 (26-46)	58 (54-64)	66 (59-73)	72 (63-76)	<0.0001
Male, n (%)	26 (41.9)	18 (69.2)	15 (71.4)	15 (62.5)	0.028
Comorbidities, n (%)					
Hypertension	0 (0)	8 (30.8)	12 (57.1)	18 (75)	<0.0001
Diabetes	0 (0)	0 (0)	8 (38.1)	11 (45.8)	<0.0001
Cardiovascular diseases	0 (0)	0 (0)	1 (4.8)	17 (70.8)	<0.0001
Respiratory diseases	5 (8.1)	2 (7.7)	3 (14.3)	1 (4.2)	0.672
Dyslipidemia	3 (4.8)	0 (0)	2 (9.5)	6 (25)	0.008
Other diseases^a^	4 (6.5)	5 (19.2)	1 (4.8)	6 (25)	0.041
Clinical symptoms, n (%)					
Fever	22 (35.5)	13 (50)	13 (61.9)	12 (50)	0.160
General symptoms^b^	23 (37.1)	10 (38.5)	9 (42.9)	13 (54.2)	0.536
Respiratory symptoms	30 (48.4)	15 (57.7)	15 (71.4)	17 (70.8)	0.143
ENT symptoms	21 (33.9)	7 (26.9)	6 (28.6)	6 (25)	0.868
Digestive symptoms	15 (24.2)	6 (23.1)	7 (33.3)	4 (16.7)	0.636
Outcomes, n (%)					
Secondary infection	3 (4.8)	6 (23.1)	6 (28.6)	12 (50)	<0.0001
Invasive mechanical ventilation	1 (1.6)	4 (15.4)	2 (9.5)	7 (29.2)	0.001
ARDS	2 (3.2)	3 (11.5)	2 (9.5)	6 (25)	0.017
Thromboembolic complications	2 (3.2)	0 (0)	1 (4.8)	1 (4.2)	0.743
Multi-organ failure	2 (3.2)	2 (7.7)	1 (4.8)	4 (16.7)	0.148
ICU admissions	6 (9.7)	9 (34.6)	11 (52.4)	20 (83.3)	<0.0001
Death	1 (1.6)	2 (7.7)	2 (9.5)	9 (37.5)	<0.0001

For the univariate analysis (Table 4), we found that the cardiovascular risk level was associated with ICU admission. In fact, compared to patients with a low level of cardiovascular risk, only those patients with a very high level of cardiovascular risk were more likely to require ICU admission [OR = 46.67, 95% CI: (11.93-182.60)]. Furthermore, patients with a very high cardiovascular risk level were still more likely to require ICU admission when compared with all other patients including those with low, moderate, or high levels of cardiovascular risk [OR = 15.96, 95% CI: (5.00-50.94)]. 

For the multivariate analysis (Table 4), we found that patients having very high cardiovascular risk levels were independently associated with ICU admission. Indeed, these patients with a very high cardiovascular risk level were more likely to require ICU admission when compared with patients with low cardiovascular risk level [OR = 11.94, 95% CI: (1.34-106.78)] and also when compared with all other patients including those with a low, moderate, or high levels of cardiovascular risk [OR = 6.42, 95% CI: (1.45-28.30)].

**Table 4:** Association of cardiovascular risk with ICU admission ^a^In this model, we included the patient's "cardiovascular risk" with four dummy variables (low level, moderate level, high level, and very high level) ^b^In this model, we included the variable "very high cardiovascular risk level" only ^c^Dizziness, myalgias, asthenia, etc. ^d^Hyperuricemia, neoplasia, smoking, obesity, etc. ICU: intensive care unit; ENT: ear, nose, and throat; OR: odds ratio; CI: confidence interval

Variables	Univariate model, OR (95% CI)	Multivariate model^a^, OR (95% CI)	Multivariate model^b^, OR (95% CI)
Demographics			
Age, years	1.08 (1.05-1.11)	1.05 (1.00-1.11)	1.07 (1.03-1.11)
Male	3.92 (1.76-8.70)	3.19 (1.05-9.69)	3.24 (1.07-9.81)
Cardiovascular risk			
Low level	1	1	
Moderate level	4.94 (1.54-15.87)	1.64 (0.34-7.98)	
High level	10.27 (3.09-34.12)	2.17 (0.32-14.68)	
Very high level	46.67 (11.93-182.60)	11.94 (1.34-106.78)	
Very high cardiovascular risk level			
No	1	1	
Yes	15.96 (5.00-50.94)		6.42 (1.45-28.30)
Comorbidities			
Hypertension	4.80 (2.15-10.71)		
Diabetes	4.03 (1.46-11.13)		
Cardiovascular diseases	13.55 (3.67-50.02)		
Respiratory diseases	2.46 (0.71-8.55)		
Dyslipidemia	1.65 (0.47-5.72)		
Other diseases^c^	3.75 (1.27-11.10)	5.95 (1.20-29.41)	6.55 (1.33-32.19)
Clinical symptoms			
Fever	2.33 (1.12-4.82)		
General symptoms^d^	1.97 (0.95-4.07)		
Respiratory symptoms	4.83 (2.08-11.21)	6.24 (1.96-19.84)	6.51 (2.06-20.58)
ENT symptoms	1.03 (0.47-2.23)		
Digestive symptoms	1.18 (0.52-2.70)		

## Discussion

In this study, we investigated the association of cardiovascular risk levels of the patients with the severity of COVID-19 disease; this is the first major study involving an African population with COVID-19. The patients' level of cardiovascular risk was assessed by the doctor who had recommended their hospitalization, following the 2019 ESC, EAS, and EASD guidelines [9,10].

Patients with a very high level of cardiovascular risk had a significantly higher rate of complications including secondary infection and ARDS. In addition, these patients also showed generally poorer clinical outcomes. More than four-fifths of them were admitted to the ICU and about one-third of them required invasive mechanical ventilation. The death rate was also higher among patients with a very high cardiovascular risk level. Interestingly, we found that a patient's very high cardiovascular risk level was strongly and independently associated with ICU admissions.

These findings are in agreement with previous reports that have linked worse outcomes in COVID-19 infection with cardiovascular risk factors such as advanced age, hypertension, and diabetes, as well as with cardiovascular diseases such as coronary heart disease, heart failure, and cerebrovascular infarction. Indeed, Wang et al. have reported higher rates of ICU admissions in patients with several comorbidities [12]. Furthermore, a recent prospective study involving an American cohort of 5,279 patients with COVID-19 has shown that advanced age followed by heart failure were the strongest risk factors for severe COVID-19 illness [13].

Similarly, a study that involved an Italian cohort of 1,591 infected patients with COVID-19 in the Lombardy region has shown that 68% of the patients admitted in ICU units were elderly individuals and had at least one comorbidity. The most common comorbidities in this study were hypertension (49%) followed by cardiovascular disease (21%) and hypercholesterolemia (18%) [14].

Moreover, Guan et al. have reported that among 1,099 patients with laboratory-confirmed COVID-19 disease from 552 hospitals in 30 Chinese provinces, hypertension and diabetes were strongly associated with admission to the ICU unit, the use of mechanical ventilation, and death as a composite endpoint [15]. Likewise, the reported mortality rate was higher in patients with underlying cardiovascular diseases than patients without comorbidities [16]. WU et al. have reported that among patients who succumbed to the disease, 10.5% had cardiovascular disease, 7.3% had diabetes, and 6% had hypertension [4]. Also, Li et al. have reported clinical characteristics of 25 death cases infected with COVID-19 pneumonia and found that among them, 64% had hypertension, 40% had diabetes, 32% had heart diseases, 20% had kidney diseases, and 16% had cerebral infarction [17]. Similarly, an Italian study that described the characteristics of patients who died as a result of COVID-19 has observed that deceased patients were predominantly elderly males with multiple comorbidities [18].

Several mechanisms may be attributed to the association of cardiovascular risk factors with worse clinical outcomes in patients with COVID-19. Patients with a higher level of cardiovascular risk may have a lower grade of vascular inflammation, which combines with the immune response induced by the virus and leads to an aggravation of the inflammatory state [19]. Secondly, the procoagulant state reported in COVID-19 may increase the risk of thromboembolic and acute cardiovascular events, by activating prothrombotic factors in atheromatous plaque [20]. Finally, previous studies have reported a significant association of myocardial injury with fatal outcomes of COVID-19 [21]. Consequently, patients with cardiovascular risk factors are more likely to have a severe form of COVID-19.

Limitations

Our study has some limitations. Firstly, it was observational by design, and hence further multicentric prospective and/or retrospective studies are required to validate our results. Secondly, only 133 patients were included in the study, and a larger study including more participants is highly recommended in order to verify our results. Thirdly, our assessment of COVID-19 severity was based on ICU admissions, and factors related to disease progression were not analyzed extensively. Finally, this was a single-center study, and this may have led to some biases in the results. Therefore, further multicenter studies are recommended to rectify this issue.

## Conclusions

This study found that an increased level of cardiovascular risk seems to be associated with the severity of COVID-19 disease. Further studies need to be conducted in order to study the mechanisms that induce and aggravate the severity of COVID-19 in patients with cardiovascular risk factors. Our findings also highlight the need for early assessment of cardiovascular risk levels in all patients with COVID-19, as well as implementing a priority vaccination strategy for patients with cardiovascular diseases.
